# Grandparent support during childhood is associated with emotional wellbeing in emerging adulthood

**DOI:** 10.3389/fpsyg.2025.1680383

**Published:** 2025-10-03

**Authors:** Jane M. Stephenson, Laura L. Carstensen

**Affiliations:** Department of Psychology, Stanford University, Stanford, CA, United States

**Keywords:** grandparent–grandchild relationships, intergenerational relationships, emerging adulthood, social support, emotional wellbeing

## Abstract

**Background:**

Due to longer life expectancies and age-related socioemotional strengths, grandparents are well-positioned to play roles that contribute positively to their grandchildren’s emotional development. Prior research shows that strong emotion regulation and social skills, as well as familial social support serves individuals well during emerging adulthood, a time when emotional wellbeing is challenged. Given this, and the fact that social and emotional skills are learned in early childhood, we reasoned that grandparental relationships in both early childhood and emerging adulthood may play an important role in the emotional wellbeing of their grandchildren in emerging adulthood.

**Methods:**

Survey of 514 emerging adults (ages 18–29) who reported on their emotional wellbeing and family relationships. Support from grandparents during childhood was reported retrospectively along with reports of current support from grandparents. Analytical methods include multiple linear regression and moderated regression.

**Results:**

Support received from grandparents during early childhood was associated with greater emotional wellbeing in emerging adulthood. This association persisted even if grandparents had died before their grandchildren reached adulthood and was not moderated by relationship quality with parents or primary caregivers. Support from grandparents in emerging adulthood was also associated with emotional wellbeing during that time, and this association was complemented by support in childhood.

**Discussion:**

Findings highlight the importance of supportive grandparent relationships for grandchildren, pointing to the possibility that support during the developmental period when children are learning to regulate emotion and navigate social situations is especially protective of emotional wellbeing in emerging adulthood. This work underscores the importance of considering multiple generations and a life course perspective when examining how familial relationships are associated with wellbeing.

## 1 Introduction

Advances in technology, medicine, and public health over the last century have resulted in significant increases in life expectancy. In the United States, life expectancy has nearly doubled in the past 120 years, increasing from 47 years in the early 1900s (Crimmins, 2015) to 78 years in 2023 (Murphy et al., 2024). Not only are people living longer, but they are also experiencing better functional (Stallard, 2016) and cognitive (Wu et al., 2017) health than past generations. Consequently, grandparents now have the opportunity to be active and healthy members of their grandchildren’s lives throughout childhood and into adulthood (Uhlenberg, 1996).

The social and emotional strengths of older adulthood position grandparents to complement parents in ways that benefit the emotional wellbeing of their grandchildren. Age-related gains in emotional and interpersonal skills, such as resilience (Almeida et al., 2023) and conflict negotiation (Blanchard-Fields et al., 2007), along with an increased preference for close relationships and meaningful experiences (Fredrickson and Carstensen, 1990), make grandparents prime candidates to model and encourage the development of emotional and social skills in their young grandchildren (Barnett et al., 2010; Rosebrook, 2002) that may benefit grandchildren’s emotional wellbeing as they navigate the challenges that come with the transition into adulthood (Goodman et al., 2015; Olsson et al., 2013).

The present study was designed to investigate the association between close relationships with grandparents during early childhood and grandchildren’s emotional wellbeing, defined as a composite of positive emotional experience, life satisfaction, flourishing, and resilience (Park et al., 2022), and mental distress, defined as symptoms of anxiety and depression, in emerging adulthood (ages 18–29). Below, we review the literature on the socioemotional strengths of older adulthood, and the socioemotional skills young children need to acquire for successful development and navigation of emotional and mental health challenges in emerging adulthood.

Older age is associated with notable improvements in social and emotional functioning and increased investment in close relationships, meaning that grandparents may be particularly well suited to support the emotional development and wellbeing of their grandchildren (Bengtson, 2001). Compared to younger people, older people experience less negative emotion (Stone et al., 2010), observed in both cross-sectional (Carstensen et al., 2000) and longitudinal research (Carstensen et al., 2011). Apart from dementias, older people have lower rates of all other forms of psychopathology (Blazer, 2003). Older adults are also more resilient in the face of stress (Almeida et al., 2023), report fewer stressors and interpersonal tensions (Birditt et al., 2005), and more effectively resolve social conflicts (Blanchard-Fields et al., 2007; Charles et al., 2009). Socioemotional Selectivity Theory (SST) offers a theoretical account of these observations. SST asserts that motivational shifts occur when time horizons shrink (Carstensen, 2006; Carstensen et al., 1999; Carstensen and Reynolds, 2023) such that emotionally meaningful goals are highly prioritized. Consequently, social preferences favor close relationships (Fredrickson and Carstensen, 1990), positive information is attended to and remembered better than negative (Charles et al., 2003; Charles and Carstensen, 2008; Isaacowitz et al., 2006), and social networks are selectively pruned to include disproportionately high numbers of emotionally close others (English and Carstensen, 2014). Older adults’ selective focus on loved ones is consistent with the grandmother hypothesis, which maintains that grandparents have a genetic stake in the wellbeing and evolutionary success of their grandchildren (Carstensen and Löckenhoff, 2003; Hawkes, 2003).

During early childhood (before age 12), children develop emotional and social skills, including self-awareness and regulation, social awareness, communication, listening, conflict negotiation, and decision making (Greenberg et al., 2017). Evidence from longitudinal research underscores the link between greater mastery of these social and emotional skills and better emotional wellbeing and fewer mental health symptoms in adulthood. One review found that self-perception, self-control, social skills, resilience, and coping in childhood were predictive of fewer symptoms of mental distress and greater life satisfaction adulthood (Goodman et al., 2015). Likewise, cooperation, confidence, and sharing in childhood have also been linked to more effective coping behaviors in adulthood (Olsson et al., 2013). Developmental research links grandparent involvement to better wellbeing outcomes for grandchildren (Sear and Coall, 2011). Children with more involved grandparents tend to exhibit fewer externalizing behaviors (Barnett et al., 2010; Tan et al., 2024). To our knowledge, however, the reach of potential emotional benefits from childhood and into young adulthood has not been explored.

Emerging adulthood (ages 18–29) is a life stage in which individuals navigate considerable uncertainty and change as they are launched from families of origin and begin to navigate life on their own (Arnett et al., 2014). The myriad emotional challenges associated with being an emerging adult in today’s world are evident in high rates of mental illness observed in this age group (Substance Abuse and Mental Health Services Administration, 2023). Young adults today experience higher rates of depression and anxiety than any other age group and higher than previous generations experienced at the same age in the 1990s and early 2000s (Udupa et al., 2023). Studies have found support for a connection between the features of emerging adulthood and struggles with emotional wellbeing and mental distress. Greater perceived instability in emerging adulthood is associated with higher levels of depressive symptoms and lower self-esteem (Luyckx et al., 2011), as are feelings of being in between childhood and adulthood (Brito and Soares, 2023). In addition to evidence that emerging adulthood is associated with higher rates of mental health problems compared to middle aged and older adults, there is evidence that rates of distress have been increasing over historical time. Compared to past generations at the same age in the 1990s and early 2000s, emerging adults today report spending more days per month in poor mental health and more emerging adults today meet the criteria for moderate to high mental distress than those in the past (Udupa et al., 2023).

Despite these challenges, there is also evidence that protective factors, including social and emotional skills learned in early childhood and ongoing family support, bolster emotional wellbeing and reduce mental distress during emerging adulthood. Social and emotional skills, including self-regulation, emotional intelligence, and wisdom, are negatively associated with depressive symptoms in emerging adulthood (Brito and Soares, 2023). In addition, social support, particularly family support, is positively associated with emotional wellbeing (Lee et al., 2018; García Mendoza et al., 2019).

Although limited, a small literature provides qualified support for the suggestion that grandparents contribute to better mental health and emotional wellbeing outcomes in young adult grandchildren. Greater affinity towards a grandparent is associated with lower depressive symptoms in young adult grandchildren, but support exchange has no association (Moorman and Stokes, 2016). Young adults who confide more in their grandparents report lower depressive symptoms compared to those who share less (Ruiz and Silverstein, 2007). While evidence suggests that support from grandparents in both childhood and emerging adulthood may have positive associations with emotional wellbeing in emerging adulthood, research has not yet explored compounding effects of grandparent support in both childhood and emerging adulthood, and whether one in the absence of another has differential associations with emotional wellbeing and mental distress.

Grandparent support may be particularly important for the emotional wellbeing of grandchildren in emerging adulthood when other sources of support, such as parents or other primary caregivers, are limited or when these relationships are strained. Past research indicates that grandparental involvement can be protective in the presence of parental separation and maternal depression (Attar-Schwartz et al., 2009; Silverstein and Ruiz, 2006; Yang and Wild, 2022), though the capacity of support from grandparents during childhood to buffer the association between poor relationship quality with parents or primary caregivers and emotional wellbeing in emerging adulthood has not been explored.

In sum, the social and emotional strengths associated with older adulthood align well with the social and emotional developmental needs of children which, when successfully acquired, may benefit their emotional wellbeing as they transition into adulthood. Due in part to the relatively recent increase in life expectancy and subsequent increased presence of grandparents in the lives of grandchildren, the potential benefits of these relationships are important to explore. Building on the existing literature, we hypothesize that socioemotional support from grandparents during early childhood is associated with better emotional wellbeing in emerging adulthood, a life stage in which healthy social and emotional functioning is critical due to the uncertainty, instability and mental health challenges that characterize this developmental period. Furthermore, we explore how associations between grandparental support and wellbeing in emerging adulthood vary based on emerging adults’ relationships with their parents or primary caregivers.

The present study was designed to test the following three hypotheses and address five research questions:

Hypothesis 1: Having a relationship with at least one grandparent is associated with better emotional wellbeing and less mental distress compared to not having a relationship with any grandparent.

Hypothesis 2: Receiving support from a close grandparent during childhood is associated with better emotional wellbeing and less mental distress in emerging adulthood.

Hypothesis 3: Receiving support from a close grandparent in emerging adulthood is associated with better emotional wellbeing and less mental distress.

Research Question 1: Are the associations between receiving support from a close grandparent in childhood, emotional wellbeing, and mental distress in emerging adulthood moderated by whether the grandparent is still living?

Research Question 2: Among emerging adults whose grandparents have died, are the associations between support from a close grandparent in childhood, emotional wellbeing, and mental distress in emerging adulthood moderated by the grandchild’s age when their grandparent died?

Research Question 3: Are the associations between receiving support from a close grandparent in emerging adulthood, emotional wellbeing, and mental distress moderated by receiving support from a grandparent in childhood?

Research Question 4: Are the associations between receiving support from a close grandparent in childhood, emotional wellbeing, and mental distress in emerging adulthood moderated by closeness with parents or primary caregivers?

Research Question 5: Are the associations between receiving support from a close grandparent in emerging adulthood, emotional wellbeing, and mental distress moderated by closeness with parents or primary caregivers?

## 2 Materials and methods

### 2.1 Study design and participants

We recruited 514 emerging adults for this study. Emerging adulthood was operationalized as 18 to 29 years of age (Arnett et al., 2014). We planned to collect a large sample that could provide sufficient power to test the moderating effect of grandparent support on the association between closeness with primary caregivers and emotional wellbeing in emerging adulthood with a small to medium effect size. To detect an effect size of 0.05, with 5% error, 80% power, and six predictors, (age, gender, race, predictor, moderator, and interaction term) we would have needed a sample size of 279. Conducting a moderation analysis only on the subset of participants who reported feeling closest to their grandparents in childhood (*n* = 305), we achieved 84% power.

Participants were recruited via the online survey platform Prolific using the platform’s standard sample distribution with the inclusion criteria that participants were located in the United States and between the ages of 18 and 29. On the platform, 167,385 users were eligible for the study based on the above criteria. A random subset of 29,574 were also notified about the study to avoid bias towards more active users. To further avoid bias in recruiting participants who had relationships with grandparents, the study title and description did not explicitly mention grandparents. Participants were told only that they were invited to participate in a study on family relationships and wellbeing. Once recruited, participants were directed to an online survey in which they gave informed consent to participate and reported their emotional wellbeing, relationships with parents or primary caregivers, and relationships with the grandparents with whom they felt closest if they had any. On average, this survey took approximately 20 min to complete. Participants were excluded from the study if they failed more than two attention checks (*n* = 47), provided a date of birth that placed them outside the age range of emerging adults (ages 18–29) despite the survey explicitly targeting those in that range (*n* = 6), or failed to answer a substantial number of questions (*n* = 12). Participants gave informed consent before taking part in the study and were compensated $3 for their time. Dta were collected between January 2025 and May 2025 and all components of the research took place in the United States. The University Institutional Review Board approved all study procedures (Protocol Number: 78081, Title: “Grandparent Relationships and Emotional Wellbeing in Young Adults”). Data, study materials, and code can be found under https://osf.io/6h8kt/?view_only=2faf738b81dd45c0a81d6976bdfb6786. Any further details about this study will be provided by the corresponding author upon request.

### 2.2 Measures

#### 2.2.1 Grandparent relationships

To assess whether participants had any relationship with a grandparent, they were first asked how many grandparents they had a relationship with at any point in their lives, even if the grandparents had since passed away (*At least one grandparent relationship* = 1, *No grandparent relationship* = 0). Those who reported no relationships were directed to the end of the survey. Those who reported at least one grandparent were asked to think about the grandparent with whom they felt closest, regardless of whether that grandparent was still living. They were then asked whether this grandparent was still living (*Living GP* = 1) or deceased (*Living GP* = 0), how old they were when this grandparent died if applicable, and the time period during which they felt closest to this grandparent: childhood (ages 0–12), adolescence (ages 13–17), early adulthood (18 - present).

All participants who reported a close relationship with a grandparent reported how often they received three forms of support from their closest grandparents during the time they felt closest to them. Those who reported that the grandparents with whom they felt closest were still living also reported the frequency with which they currently received each form of support. Support was assessed with three items: how much participants share(d) what is(was) going on in their lives with their closest grandparents, how much they receive(d) emotional support from their closest grandparents, and how much they receive(d) advice from their closest grandparents (Fingerman et al., 2016; Umberson, 1992; Vaux and Harrison, 1985). All items were reported on a five-point scale from *none* (1) to *a great deal* (5). Because all forms of support in each life stage were moderately correlated and had high internal consistency,^1^ composite scores for support received from grandparents in childhood and support received from grandparents in emerging adulthood were computed by averaging participants’ ratings of each form of support (sharing, emotional support, advice) during each life stage.

#### 2.2.2 Primary caregiver relationships

Participants reported subjective closeness with a maximum of two primary caregivers. For each primary caregiver identified (e.g., mother, father, stepmother, stepfather, grandparent, etc.), participants report how emotionally close they felt to each primary caregiver from *not at all close* (1) to *very close* (5). Closeness scores for both caregivers were averaged to create a single measure.

#### 2.2.3 Emotional wellbeing and mental distress

Participants reported the degree to which they experienced 19 emotions within the past 2 weeks on a scale from *not at all* (1) to *extremely* (7) (Carstensen et al., 2011). Positive emotional experience was based on the average of happiness, joy, contentment, excitement, pride, accomplishment, interest, and amusement (Cronbach’s Alpha = 0.90), and negative experience was based on the average of anger, sadness, fear, disgust, guilt, embarrassment, shame, anxiety, irritation, frustration, and boredom (Cronbach’s Alpha = 0.92).

The Satisfaction With Life Scale (Diener et al., 1985) consists of five statements (e.g., “In most ways, my life is close to ideal”) that are rated on a scale from *strongly disagree* (1) to *strongly agree* (7), the scores for which are then averaged (Cronbach’s Alpha = 0.91).

The Flourishing Scale (Diener et al., 2010) includes eight statements (e.g., “I lead a purposeful and meaningful life”) that are rated on a seven-point scale from *strongly disagree* (1) *strongly agree* (7). The responses are summed to yield a total score (Cronbach’s Alpha = 0.92).

The abbreviated Connor-Davidson Resilience Scale (Connor and Davidson, 2003; Vaishnavi et al., 2007) consists of two items (“I am able to adapt when changes occur” and “I tend to bounce back after illness, injury, or other hardships”), the truthfulness of which is rated on a five-point scale from *not true at all* (1) to *true nearly all of the time* (5). The scores for both items are summed (items were correlated at *r* = 0.49).

Finally, the Four-Item Patient Health Questionnaire (PHQ; Kroenke et al., 2009) assesses symptoms of depression and anxiety by instructing participants to report how often in the past 2 weeks they have been bothered by each of four items (e.g., “Little interest or pleasure in doing things.”) on a scale from *not at all* (1) to *nearly every day* (4); scores on this measure were summed to yield a total score (Cronbach’s Alpha = 0.86).

To reduce the number of variables, an exploratory factor analysis was conducted on the above measures. The analysis yielded two factors with eigenvalues greater than 1.0: one that consisted of positive emotional experience, flourishing, satisfaction with life, and resilience, which will be referred to as emotional wellbeing (*M* = 0, *SD* = 0.96, range: −3.17–1.68, omega total = 0.91, omega general = 0.61), and one that included negative emotional experience and PHQ score, which will be referred to as mental distress (*M* = 0, *SD* = 1.00, range: −1.45–2.78, omega total = 0.91, omega general = 0.82). The factors for emotional wellbeing and mental distress were correlated (*r =* −0.62). These two factor scores, emotional wellbeing and mental distress, served as the dependent variables for this study.

### 2.3 Analytic strategy

All analyses were computed using R (R Core Team, 2025). We tested our first hypothesis by running a t-test of mean differences in emotional wellbeing and mental distress between participants who did and did not have a relationship with a grandparent. To test our second and third hypotheses, we ran linear models^2^ of emotional wellbeing and mental distress regressed on the composite of support received from grandparents in childhood and the composite of support received from grandparents in the present, respectively.

To explore Research Question 1, we analyzed models of emotional wellbeing and mental distress regressed on support received from grandparents in childhood with whether the grandparent was deceased as a moderator. Likewise, to explore Research Question 2, we created linear models of support received from grandparents in childhood predicting emotional wellbeing and mental distress with participants’ age at their grandparent’s death as a moderator. To explore Research Question 3, we regressed emotional wellbeing and mental distress on an interaction between support received from grandparents in childhood and support received from grandparents in the present. We explored our fourth research question by first running a linear model of support received from grandparents in childhood predicting emotional wellbeing and mental distress while controlling for closeness with primary caregivers. Then, we ran a linear model with support received from grandparents in childhood interacting with closeness with primary caregivers to predict emotional wellbeing and mental distress. Likewise, for Research Question 5, we regressed emotional wellbeing and mental distress on present support while controlling for closeness with primary caregivers and then ran models of emotional wellbeing and mental distress with present support as a predictor and closeness with primary caregivers as a moderator.

## 3 Results

Of the participants who took part in the study, the average age was 24.43 years (*SD* = 2.97). Fifty-eight percent (*n* = 299) of the participants identified as female, 40% (*n* = 205) male, and 2% (*n* = 10) non-binary or elected not to answer. Fifty-three percent (*n* = 272) of participants identified as White or Caucasian, 35% (*n* = 178) as Black or African American, 6% (*n* = 32) Asian, 4% (*n* = 21) mixed race, 1% (*n* = 3) American Indian/Native American or Alaska Native, and 1% (*n* = 7) reported that they identified with a race not listed. Further descriptive statistics of demographic covariates and study variables are presented in Table 1.

**Table 1:** Descriptive statistics.

Variables	%	*n*
Gender		
Female	58	299
Male	40	205
Other	2	10
Race		
Black	35	178
White	53	272
Other	12	63
GP Relationship	92	475
Living GP	47	142

Variables	*M* (*SD*)	Range
Age	24.43 (2.97)	18–29
Emotional wellbeing	0.00 (0.96)	−3.17 - 1.68
Mental distress	0.00 (1.00)	−1.45 - 2.78
PC closeness	3.86 (1.13)	1–5
Age at GP’s death	15.69 (5.64)	1–28
Support in childhood	3.78 (0.99)	1–5
Present support	3.02 (1.17)	1–5

GP Relationship: Whether participants reported at least one relationship with a grandparent at some point in their life (1 = yes, 0 = no). Living GP: Whether the closest grandparent is still living (1 = still living, 0 = deceased). Age at GP’s Death: Participants age when grandparent died. Support in Childhood: Composite of support from grandparents during childhood. Present Support: Composite of support received from grandparents in the present.

### 3.1 Presence of a grandparent and wellbeing and distress in emerging adulthood

**Figure 1:**
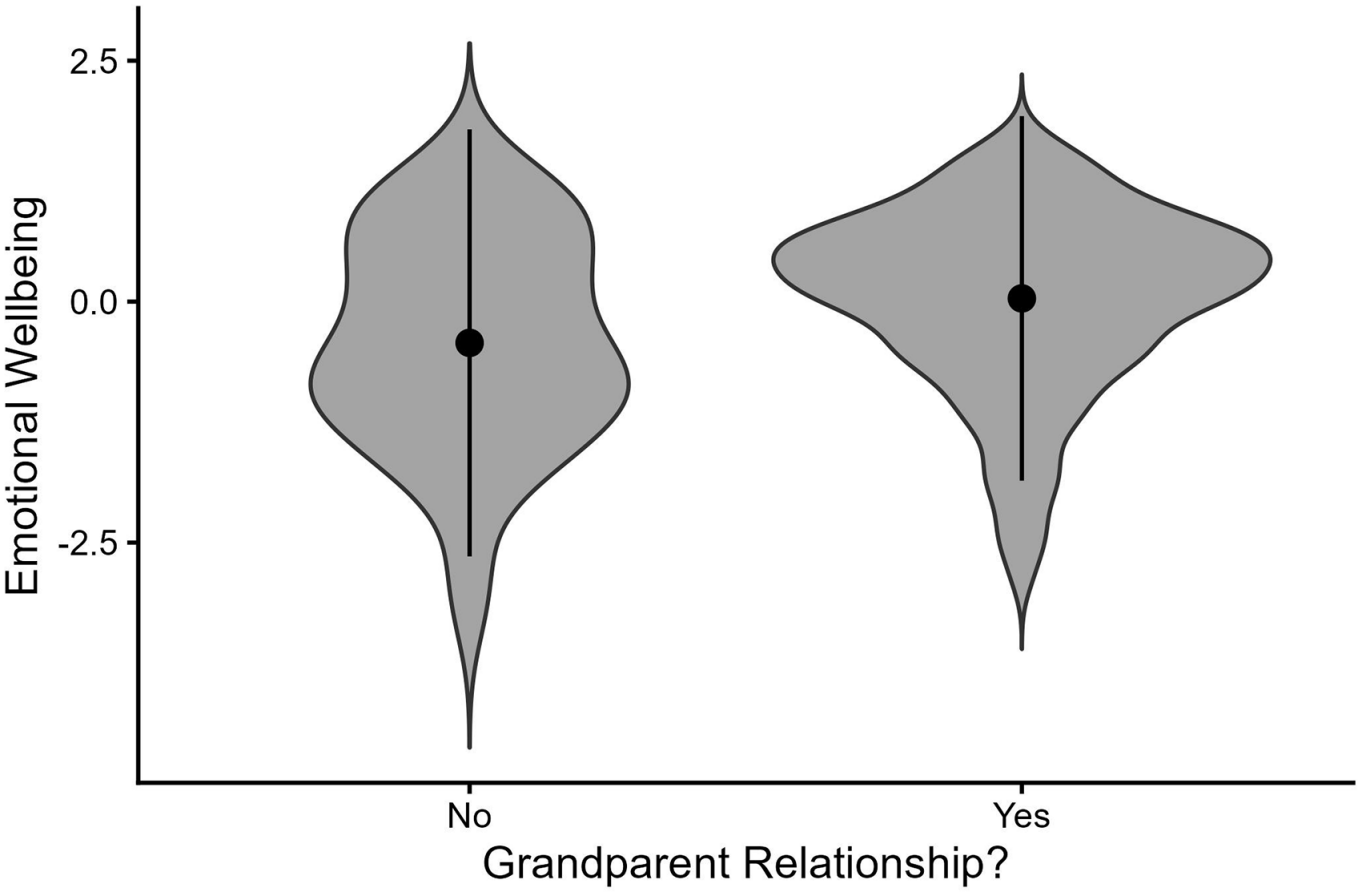
Emerging adults with grandparent relationships report better emotional wellbeing compared to those with no grandparents relationships. Black dots denote average of each group, lines denote standard deviations. Shaded violin plot represents distribution of responses. Full range of Emotional Wellbeing spans from −3.17 to 1.68.

Of the 514 participants, 92% (*n* = 475) reported a relationship with at least one grandparent at some point in their life. On average, participants with a relationship with at least one grandparent reported greater emotional wellbeing (*M* = 0.03) than did those who had no relationships with any grandparents [*M* = −0.43, *t*(40.193) = −2.47, *p* = 0.02; Cohen’s *d* = 0.48]. A visualization of group means, standard deviations, and distribution of emotional wellbeing scores for participants who did and did not report having a relationship with a grandparent can be found in Figure 1. Having a relationship with a grandparent was not associated with mental distress [*M* = 0.002 and *M* = −0.02, respectively, *t*(41.603) = −0.14, *p* = 0.89; Cohen’s *d* = 0.02].

### 3.2 Support in childhood, wellbeing and distress in emerging adulthood

Of the participants who reported a relationship with a grandparent, 59% (*n* = 305) reported that they felt closest to their grandparent in childhood, 21% (*n* = 110) in adolescence, and 13% (*n* = 66) in emerging adulthood. All subsequent analyses are based on the 305 participants who reported that they felt closest to their closest grandparent during childhood, as support provided to grandchildren during childhood is of particular interest in the present study.

**Figure 2:**
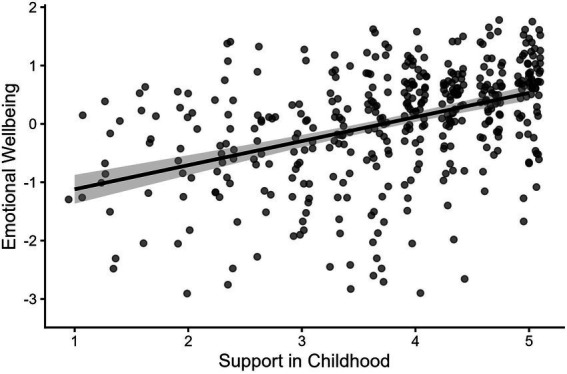
Support from grandparents during childhood is associated with better emotional wellbeing in emerging adulthood. Support in Childhood = Composite of amount of support received from grandparents during childhood (1 = *none*, 5 = *a great deal*). Full range of Emotional Wellbeing spans from −3.17 to 1.68. Points have been jittered by 0.1 on both axes for readability.

Receiving support from grandparents in childhood was positively correlated with emotional wellbeing [*r*(303) = 0.38, *p* < 0.001] and support from grandparents in childhood was positively associated with emerging adult’s emotional wellbeing in a linear model (*Est* = 0.34, *SE* = 0.05, *p* < 0.001; Table 2, Model 1; Figure 2). Support from grandparents during childhood was significantly correlated with mental distress [*r*(303) = −0.13, *p* = 0.03], but did not significantly predict mental distress in a linear model [*Est* = −0.10, *SE* = 0.06, *p* = 0.08]. Because there was no significant association, interactions predicting mental distress were not examined.

**Table 2:** Result from linear models of support in childhood and interactions with grandparent status and exposure predicting emotional wellbeing.

Variables	Model 1 (*N* = 305)		Model 2 (*N* = 305)		Model 3 (*N* = 305)	
	*Est*	*SE*	*Est*	*SE*	*Est*	*SE*
Intercept	−1.28**	0.48	0.09	0.45	0.20	0.16
Support in childhood	0.34***	0.05	0.36***	0.07	0.41***	0.09
Living GP			−0.05	0.11		
Support in childhood × Living GP			−0.05	0.11		
Age at GP death					0.01	0.01
Support in childhood x Age at GP death					0.01	0.01
*R^2^*	0.17		0.17		0.23	

Est. = unstandardized estimate, SE = standard error. Support in Childhood = Composite of support from grandparents during childhood. Living GP: Whether the closest grandparent is still living (1 = still living, 0 = deceased). Age at GP’s Death: Participants age when grandparent died. Multiple R-squared values for each model are included. *p < 0.05. **p < 0.01. ***p < 0.001.

#### 3.2.1 Support from deceased vs. living grandparents and wellbeing

**Figure 3:**
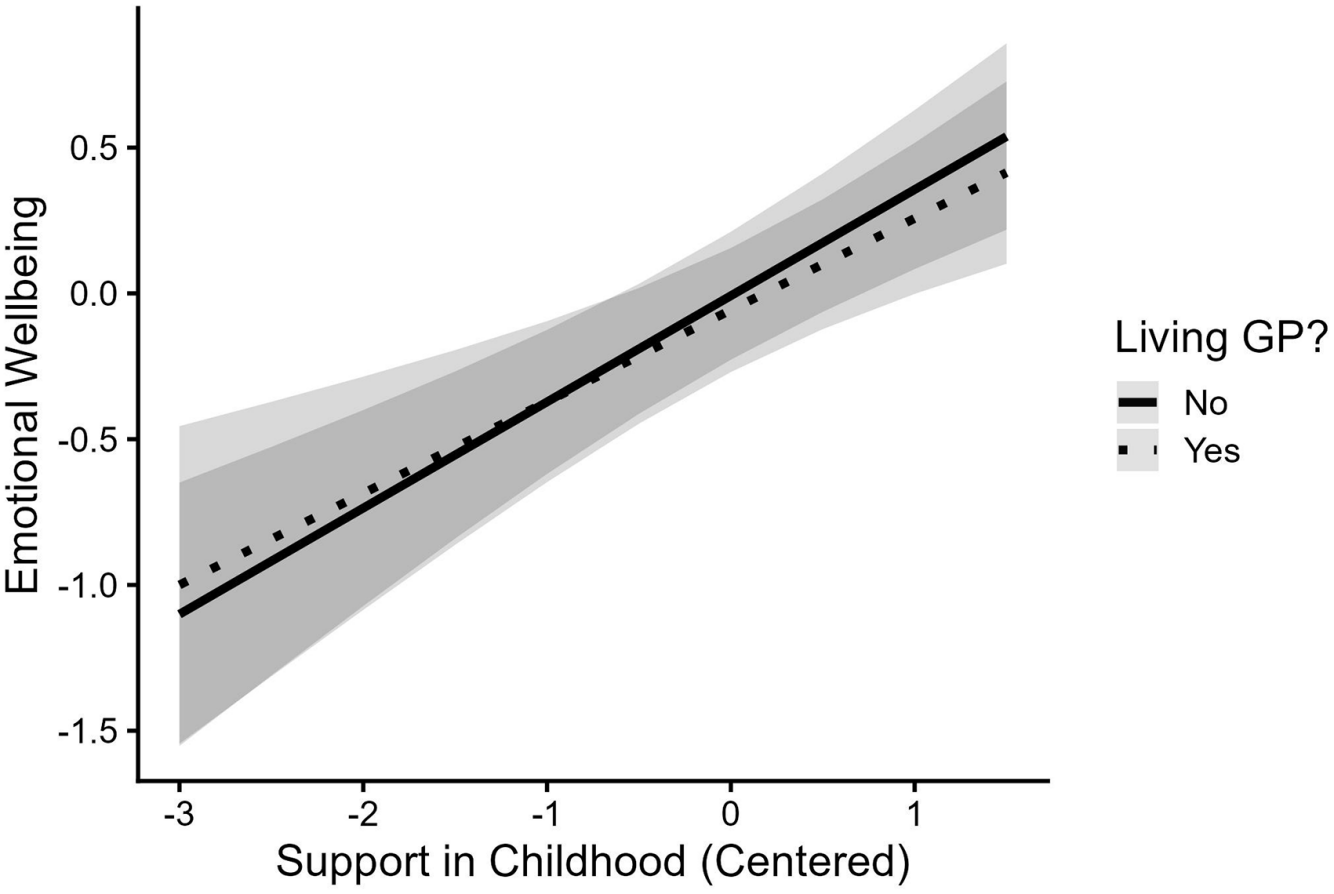
Support from grandparents during childhood is associated with better emotional wellbeing in emerging adulthood regardless of whether grandparent is still living. Support in Childhood = Composite of amount of support received from grandparents during childhood. Living GP = Whether grandparent is still living when grandchild is in emerging adulthood. Full range of Emotional Wellbeing spans from −3.17 to 1.68.

**Figure 4:**
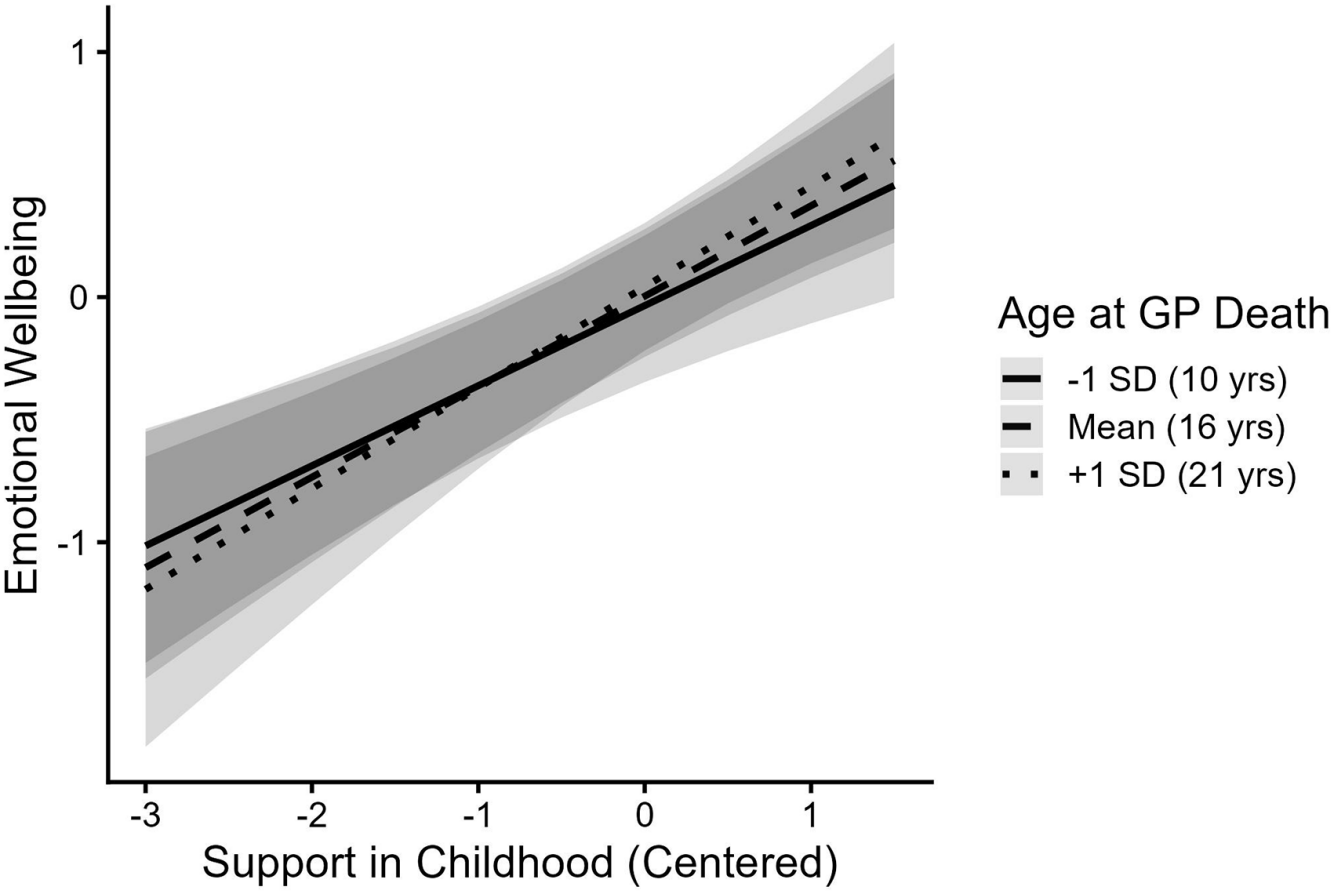
Support from grandparents during childhood is associated with better emotional wellbeing in emerging adulthood regardless of grandchild’s age when grandparent passed. Support in Childhood = Composite of amount of support received from grandparents during childhood. Age at GP Death = Grandchild’s age when grandparent died. Full range of Emotional Wellbeing spans from −3.17 to 1.68.

Out of the 305 participants who report feeling closest to their grandparents in childhood, 53 % (*n* = 163) reported that their closest grandparent was deceased. Emerging adults whose closest grandparents were still living (*M* = −0.05) did not differ in emotional wellbeing from those whose closest grandparents were deceased (*M* = 0.04, *t*(280.61) = 0.81, *p* = 0.42). Grandparent status (alive or deceased) did not moderate the association between support from grandparents during childhood and emotional wellbeing (Table 2, Model 2; Figure 3). Participants’ age at the death of their closest grandparents also did not moderate the association between receiving support from grandparents during childhood and emotional wellbeing (Table 2, Model 3; Figure 4).

### 3.3 Current support and wellbeing

**Figure 5:**
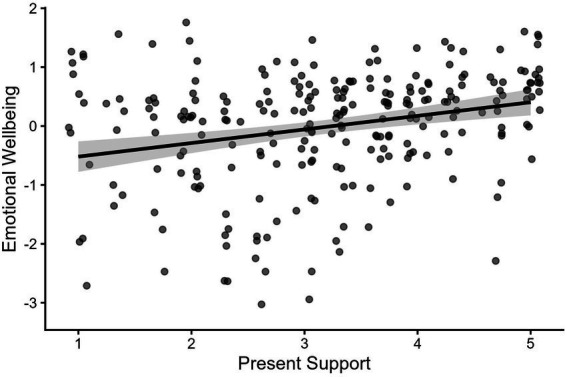
Support from grandparents during emerging adulthood is associated with better emotional wellbeing. Present Support = Composite of amount of support received from grandparents during emerging adulthood (1 = *none*, 5 = *a great deal*). Full range of Emotional Wellbeing spans from −3.17 to 1.68. Points have been jittered by 0.1 on both axes for readability.

Among the 142 participants whose closest grandparents were still living, scores on support received from grandparents in the present were significantly correlated with emotional wellbeing [*r*(140) = 0.31, *p* < 0.001] and present support from grandparents significantly predicted emotional wellbeing in a linear model (*Est* = 0.28, *SE* = 0.08, *p* = 0.001; Figure 5). Present support from grandparents was not significantly correlated with mental distress [*r*(140) = −0.11, *p* = 0.18] and did not predict mental distress in a linear model (*Est* = −0.12, *SE* = 0.08, *p* = 0.14). Consequently, interactions with mental distress as an outcome were not analyzed.

### 3.4 Support in childhood, current support, wellbeing and distress

Receiving support from grandparents in childhood was highly correlated with receiving support from grandparents in emerging adulthood (*r* = 0.75). Neither construct significantly predicted emotional wellbeing when they were included in the same model (Table 3, Model 2).

**Table 3:** Results from linear models of present support, additive and interacting effects of childhood and present support predicting emotional wellbeing.

Variables	Model 1 (*N* = 142)		Model 2 (*N* = 142)		Model 3 (*N* = 142)	
	*Est*	*SE*	*Est*	*SE*	*Est*	*SE*
Intercept	−1.16	0.76	−1.66*	0.82	−0.28	0.67
Present support	0.28***	0.08	0.14	0.12	0.03	0.12
Support in childhood			0.21	0.14	0.46**	0.16
Present support × Support in childhood					0.23**	0.08
*R^2^*	0.11		0.13		0.18	

Est. = unstandardized estimate, SE = standard error. Present Support = Composite of support received from grandparents in the present, Support in Childhood = Composite of support from grandparents during childhood. *p < 0.05. **p < 0.01. ***p < 0.001.

**Figure 6:**
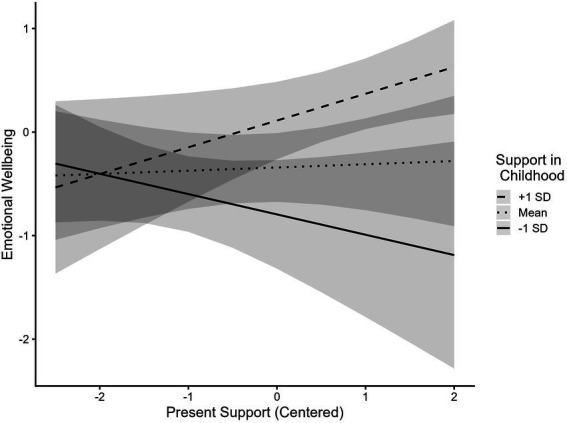
Support from grandparents in emerging adulthood is positively associated with emotional wellbeing when support in childhood is high. Present Support = Composite of amount of support received from grandparents during emerging adulthood. Support in Childhood = Composite of amount of support received from grandparents during childhood. Both Composite of support received from grandparents in the present and the composite of support from grandparents during childhood were centered. Full range of Emotional Wellbeing spans from −3.17 to 1.68.

There was a significant interaction between receiving support from grandparents during childhood and receiving support from grandparents in the present in predicting emotional wellbeing (Table 3, Model 3): Support from grandparents during childhood was positively associated with emotional wellbeing only when present support from grandparents was at mean levels (*Est* = 0.42, *SE* = 0.15, *p* = 0.01) or higher (+1 SD: *Est* = 0.69, *SE* = 0.21, *p* < 0.001), but not when present support from grandparents was below mean levels (−1 SD: *Est* = 0.15, *SE* = 0.14, *p* = 0.28). To further probe this interaction, we analyzed the simple slopes decomposed in the opposite direction as well (Figure 6). Present support from grandparents was significantly associated with emotional wellbeing only when support from grandparents during childhood was high (+1 SD: *Est* = 0.25, *SE* = 0.12, *p* = 0.04), but not when support from grandparents during childhood was at mean levels (*Est* = 0.03, *SE* = 0.12, *p* = 0.79) or below (−1 SD: *Est* = −0.19, *SE* = 0.16, *p* = 0.25).

### 3.5 Parent relationships

We ran all the above analyses controlling for participants’ self-reported closeness with their parents^3^ or primary caregivers (Table 4). Given that there were no significant associations between grandparent support and mental distress, we did not rerun those analyses. Closeness with primary caregivers was significantly correlated with emotional wellbeing [*r*(512) = 0.42, *p* < 0.001], mental distress [*r*(512) = −0.26, *p* < 0.001], support from grandparents during childhood [*r*(303) = 0.40, *p* < 0.001], and present support from grandparents [*r*(140) = 0.46, *p* < 0.001].

**Table 4:** Models of emotional wellbeing controlling for primary caregiver closeness.

Variables	Model 1 (*N* = 511)		Model 2 (*N* = 305)		Model 3 (*N* = 142)		Model 4 (*N* = 142)	
	*Est*	*SE*	*Est*	*SE*	*Est*	*SE*	*Est*	*SE*
Intercept	−1.76***	0.37	−2.12***	0.48	−1.38***	0.37	−1.80	0.76
PC closeness	0.32***	0.04	0.28***	0.05	0.28***	0.08	0.29***	0.08
GP relationship	0.24	0.15						
Support in childhood			0.22***	0.06			0.46**	0.15
Present support					0.14	0.08	−0.09	0.12
Support in childhood × Present support							0.21**	0.08
*R^2^*	0.21		0.25		0.18		0.25	

Est. = unstandardized estimate, SE = standard error. PC Closeness = Average of closeness scores for parents or primary caregivers. GP Relationship = Whether participants reported at least one relationship with a grandparent at some point in their life (1 = yes, 0 = no). Present Support = Composite of support received from grandparents in the present. Support in Childhood = Composite of support from grandparents during childhood. *p < 0.05. **p < 0.01. ***p < 0.001.

When controlling for primary caregiver closeness, there was no longer a significant difference in emotional wellbeing between participants who did and did not have a relationship with a grandparent while they were growing up (Table 4, Model 1). However, the association between support from grandparents during childhood and emotional wellbeing in emerging adults remained significant when primary caregiver closeness was added to the model (Table 4, Model 2). The association between present support from grandparents and emotional wellbeing was not significant after controlling for primary caregiver closeness (Table 4, Model 3), but the interaction between support from grandparents during childhood and support in the present remained significant (Table 4, Model 4).

**Figure 7:**
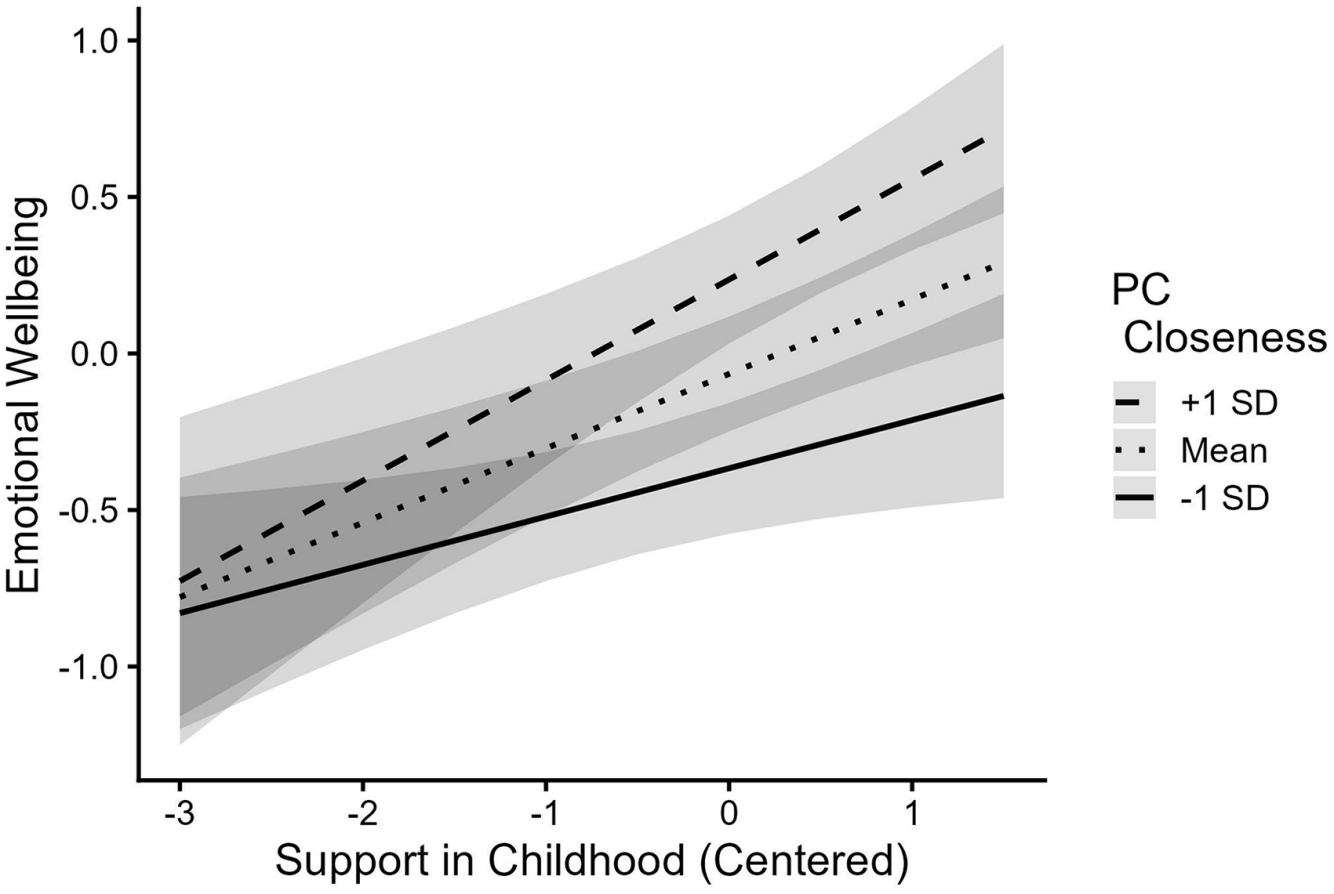
Childhood support is associated with better emotional wellbeing at all levels of primary caregiver closeness. Support in Childhood = Composite of amount of support received from grandparents during childhood. PC Closeness = Average of emerging adult’s rating of emotional closeness to two primary caregivers. Both the composite of support from grandparents during childhood and primary caregiver closeness were centered. Full range of Emotional Wellbeing spans from −3.17 to 1.68.

We also evaluated whether the associations between support from grandparents during childhood and support in the present and emotional wellbeing were significantly moderated by participants’ closeness with primary caregivers. The positive association between support from grandparents during childhood and emotional wellbeing in emerging adulthood was not moderated by participants’ closeness with their primary caregivers (Table 5, Model 1). At all levels of parent/primary caregiver closeness, higher scores on support from grandparents during childhood were associated with better emotional wellbeing (Figure 7).

**Table 5:** Models of emotional wellbeing with grandparent support moderated by primary caregiver closeness.

Variables	Model 1 (*N* = 142)		Model 2 (*N* = 142)	
	*Est*	*SE*	*Est*	*SE*
PC closeness	0.30***	0.05	0.38***	0.09
Support in childhood	0.24***	0.06		
PC closeness × Support in childhood	0.08	0.04		
Present support			0.14	0.08
PC closeness× Present support			0.15*	0.06
*R^2^*	0.26		0.23	

Est. = unstandardized estimate, SE = standard error. PC Closeness = Average of closeness scores for primary caregivers. Support in Childhood = Composite of support from grandparents during childhood. Present Support = Composite of support received from grandparents in the present. *p < 0.05. **p < 0.01. ***p < 0.001.

**Figure 8:**
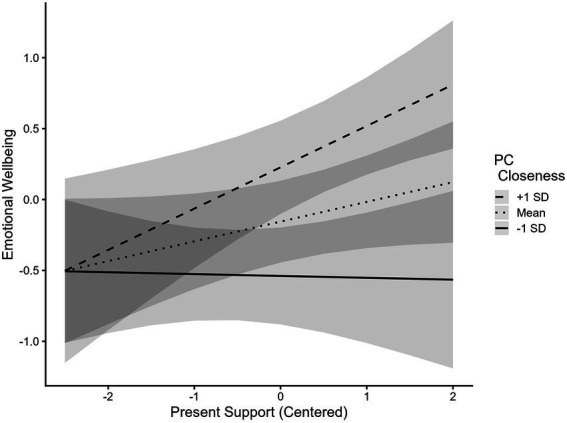
Association between present support and emotional wellbeing is moderated by primary caregiver closeness. Present Support = Composite of amount of support received from grandparents during emerging adulthood. PC Closeness = Average of emerging adult’s rating of emotional closeness to two primary caregivers. Both the composite of support received from grandparents in the present and PC Closeness were centered. Full range of Emotional Wellbeing spans from −3.17 to 1.68.

The association between present support from grandparents and emotional wellbeing was significantly moderated by participants’ closeness with their primary caregivers (Table 5, Model 2). The simple slopes of this moderation are plotted in Figure 8. At levels of closeness with primary caregivers one standard deviation below the mean and at mean levels, the association between support from grandparents in the present and wellbeing was not significant (−1 SD: *Est* = −0.05, *SE* = 0.11, *p* = 0.67; Mean: *Est* = 0.13, *SE* = 0.08, *p* = 0.13). For participants with levels of closeness with primary caregivers one standard deviation above mean levels, support from grandparents was significantly associated with emotional wellbeing (*Est* = 0.30, *SE* = 0.10, *p* < 0.01).

## 4 Discussion

This study was novel in examining how specific aspects of the grandparent-grandchild relationship (support provision) are associated with emerging adult grandchildren’s emotional wellbeing when support is provided at theoretically relevant points in emotional and social development (childhood and emerging adulthood).

### 4.1 Grandparent support

Our first hypothesis, that emerging adults who have relationships with their grandparents have better emotional wellbeing and less mental distress than those who do not, was partially supported. Participants who reported having had at least one relationship with a grandparent while growing up reported a significantly higher mean score for emotional wellbeing than participants who did not have a relationship. Receiving greater social support from grandparents during childhood and in the present was associated with better emotional wellbeing in emerging adulthood, supporting our second and third hypotheses. These findings add to the burgeoning body of research suggesting that grandparent relationships can play a role in the emotional wellbeing of young adults (Moorman and Stokes, 2016; Ruiz and Silverstein, 2007), which is important more so now than ever before as emerging adults face a multitude of challenges to their emotional wellbeing and mental health (Udupa et al., 2023).

Prior research has found that closeness between grandchildren and grandparents is relatively stable during grandchildren’s transition to adulthood (Wetzel and Hank, 2020). Our findings of a strong correlation between childhood and present support as well as a significant interaction between support in childhood and support in emerging adulthood (Research Question 3) indicate that support from grandparents also remains relatively stable throughout this transition, though shifts in support provision from childhood to emerging adulthood appear to have differential associations with emotional wellbeing. Overall, these results underscore that a life course perspective (Elder, 1998) of the dynamic nature of relationships should be taken when assessing how relationships are associated with wellbeing.

### 4.2 Grandparent death

Research has found that the death of a grandparent affects emerging adults deeply (Manoogian et al., 2018), but relationships and the resources they provide for supporting emotional wellbeing can persist beyond death (Klass et al., 2014). Our findings from exploring Research Questions 1 and 2 support this literature, indicating that grandparent relationships have a lasting impact on emerging adult grandchildren’s emotional wellbeing, even after grandparents have passed away.

### 4.3 Closeness with primary caregivers and support from grandparents

Past research has noted that in the presence of cohesive nuclear families, grandparent relationships may be redundant (Attar-Schwartz et al., 2009). However, because support from grandparents in childhood predicted emotional wellbeing during emerging adulthood independent of closeness with primary caregivers, results from this study indicate that grandparents may make unique contributions to their grandchildren’s emotional development and later emotional wellbeing. Findings from models of wellbeing with grandparent support interacting with parental closeness (Research Questions 4 and 5) indicate that while support from grandparents in emerging adulthood may not be able to compensate for the detriments of having poor relations with one’s primary caregivers during that time, support from a grandparent during a critical developmental period such as childhood may imbue grandchildren with certain protective factors that moderate the negative association between poor primary caregiver relationships and emotional wellbeing in emerging adulthood. These findings align with past research that suggests that grandparent involvement is protective in high risk situations (Silverstein and Ruiz, 2006; Yang and Wild, 2022), but adds a novel component by examining how differences in the timing of support are related to emotional wellbeing. Further research on the mechanisms through which grandparent support in childhood confers protective effects on the emotional wellbeing of grandchildren in emerging adulthood is needed.

### 4.4 Limitations and future directions

We acknowledge that the potential reliability of retrospective accounts from childhood cannot be established. However, previous research suggests that retrospective reports, when anchored to specific time points, tend to be accurate (Brewin et al., 1993) and have been used in similar studies (MaloneBeach et al., 2018). Accuracy aside, we expect that subjective reports reflect the strength of the relationship. Nonetheless, future research using longitudinal data on how grandparent-grandchild relationships evolve from childhood to emerging adulthood could strengthen the findings herein.

The motivation for this research was also predicated on the idea that grandparents utilize their age-based social and emotional strengths to impart to their young grandchildren the social and emotional skills necessary for successful development. More fine-grained questions about grandchild-grandparent interaction in childhood (e.g., “How often did your grandparent give you advice about an interpersonal problem?” or “How often did they help you see things differently when you were upset about something?”) would better capture whether this transmission of skills occurs or if the relationship between support from grandparents in childhood and emotional wellbeing later in life operates through alternative mechanisms. Finally, this research is limited to the cultural context in which the data were collected. The role of grandparents as well as their engagement with their grandchildren varies across cultural contexts (Duflos and Giraudeau, 2022). As such, the findings from this study may only be generalizable to the United States. Even among individuals living in the United States, the nature of grandparent-grandchild relationships may vary by the cultural background(s) of those within the relationship. Future research should explore cross-cultural variations in this association.

## 5 Conclusion

Once rare, increases in life expectancy have made grandparents a normative part of families and subsequently increased the need to understand the resulting developmental implications. This study sought to investigate potential benefits of grandparent relationships with their grandchildren. We found that social support provided by close grandparents during early childhood was associated with emotional wellbeing in emerging adulthood and that this association persisted beyond a grandparent’s death and even in the presence of poor parent/primary caregiver relationships. These findings support a multigenerational approach to interventions aimed at fostering the emotional wellbeing of emerging adults.

## Data Availability

The raw data supporting the conclusions of this article will be made available by the authors, without undue reservation.

## Footnotes

^1^Cronbach’s Alpha = 0.84 for items in support received from grandparents in childhood, 0.90 for items in support received from grandparents in the present.

^2^All models included participant age, gender, and race as covariates.

^3^Ninety-one percent of primary caregivers reported were mothers or fathers.
